# MRI management of focal liver lesions: what a beginner cannot fail to know

**DOI:** 10.3389/fonc.2025.1630424

**Published:** 2025-10-07

**Authors:** Vincenza Granata, Roberta Fusco, Igino Simonetti, Maria Giovanna Riga, Giuseppe Pellegrino, Serena Carriero, Michele Ahmed Antonio Karaboue, Gianpaolo Carrafiello, Antonella Petrillo, Francesco Izzo

**Affiliations:** ^1^ Division of Radiology, “Istituto Nazionale Tumori IRCCS Fondazione Pascale – IRCCS di Napoli”, Naples, Italy; ^2^ Department of Radiology, University of Padova, Padova, Italy; ^3^ Diagnostic and Interventional Radiology, Università degli Studi di Milano, Milan, Italy; ^4^ Diagnostic and Interventional Radiology Department, IRCCS Ca’ Granda Fondazione Ospedale Maggiore Policlinico, Milan, Italy; ^5^ Department of Clinical and Experimental Medicine, Section of Legal Medicine, University of Foggia, Foggia, Italy; ^6^ Division of Epatobiliary Surgical Oncology, Istituto Nazionale Tumori IRCCS Fondazione Pascale—IRCCS di Napoli, Naples, Italy

**Keywords:** liver focal lesion, MRI, DWI, contrast agents, artificial intelligence, radiomics

## Abstract

Magnetic resonance imaging (MRI) is currently recognized as the most suitable diagnostic tool for the detection and characterization of focal liver lesions. The combination of morphological and functional data allows, in different clinical scenarios, high diagnostic performance in characterizing even very small lesions, thereby improving patient management while reducing costs and examination time. Despite this premise, MRI should not be prescribed for all patients with focal liver lesions. Indications must be clearly understood, and the individual characteristics of each patient must be considered. For different clinical scenarios, depending on the presence of extrahepatic malignancy or known liver disease, MRI with contrast agents represents a useful diagnostic tool, although the choice will also depend on operator experience, technology availability, and patient-specific characteristics. A standard protocol should include conventional sequences: T2-weighted (T2W) sequences, T2W sequences with fat suppression (FS), and in-phase and opposed-phase gradient-echo T1 sequences, along with functional sequences. Among functional techniques, diffusion-weighted imaging (DWI) is mandatory, particularly for detecting very small lesions; however, diffusion restriction does not necessarily indicate malignancy. Contrast-enhanced MRI remains the cornerstone of liver MRI, especially for lesion categorization. Contrast agents can be classified as non-specific agents, which distribute into vascular and extracellular extravascular spaces, and specific agents, which are taken up by hepatic cells (Kupffer cells or hepatocytes). The abbreviated protocol concept is based on the premise that, within a shorter examination time, it is possible to acquire the essential information needed for patient management using only selected sequences from a standard protocol. Radiomics has emerged as a promising tool in liver oncology, particularly for evaluating colorectal liver metastases. To fully realize the clinical value of radiomics, it is essential to overcome several methodological hurdles, including the standardization of image acquisition and analysis workflows and rigorous validation across large and diverse patient cohorts. The aim of this review, designed for beginners in liver MRI, is to provide a comprehensive overview of the management of focal liver lesions, with a focus on acquisition protocols (including abbreviated protocols), contrast media, and reporting strategies to ensure accurate lesion characterization.

## Background

Magnetic resonance imaging (MRI) is currently recognized as the most suitable diagnostic tool for the detection and characterization of focal liver lesions (1–4). The combination of morphological and functional data allows, in different clinical scenarios, high diagnostic performance in characterizing even very small lesions (5–9), thereby improving patient management and reducing costs and time associated with inconclusive diagnostic tests (10).

Several authors have demonstrated that, in the evaluation of liver metastases, MRI not only has high diagnostic accuracy in detection (1, 2) and characterization (11–13), but also, thanks to its ability to provide functional data, enables risk stratification and appropriate patient management according to different subsets of lesions (14–17). Furthermore, in the characterization of hepatocellular carcinoma (HCC), MRI is considered the diagnostic tool of first choice, as suggested by the 2018 version of the Liver Imaging Reporting and Data System (LI-RADS) (18). A critical milestone was recently achieved with the integration of LI-RADS into the American Association for the Study of Liver Diseases (AASLD) 2018 HCC clinical practice guidance (18). Beyond HCC, MRI has also demonstrated accurate performance in the characterization of cholangiocarcinoma (19) and in the management of patients at risk for this disease (20–22).

Despite these advantages, MRI should not be prescribed for all patients. It remains an expensive examination, both in terms of cost and time. Considering the need for optimized resource use and the environmental implications of medical imaging, radiology practice must increasingly focus on sustainability and minimizing social impact (23–26). Therefore, MRI should be performed only for appropriate candidates, avoiding cases where it may represent a waste of resources. Of equal importance is the use of the most suitable contrast medium in relation to the clinical question—an aspect that remains the responsibility of the radiologist rather than the prescribing physician.

The aim of this narrative review is to critically analyze the essential aspects that a beginner in liver MRI must understand, including acquisition protocols, contrast media, and the ability to report all relevant data to characterize focal liver lesions.

## Appropriateness and indications for liver MRI examination

### Incidental liver lesion

**Figure 1:**
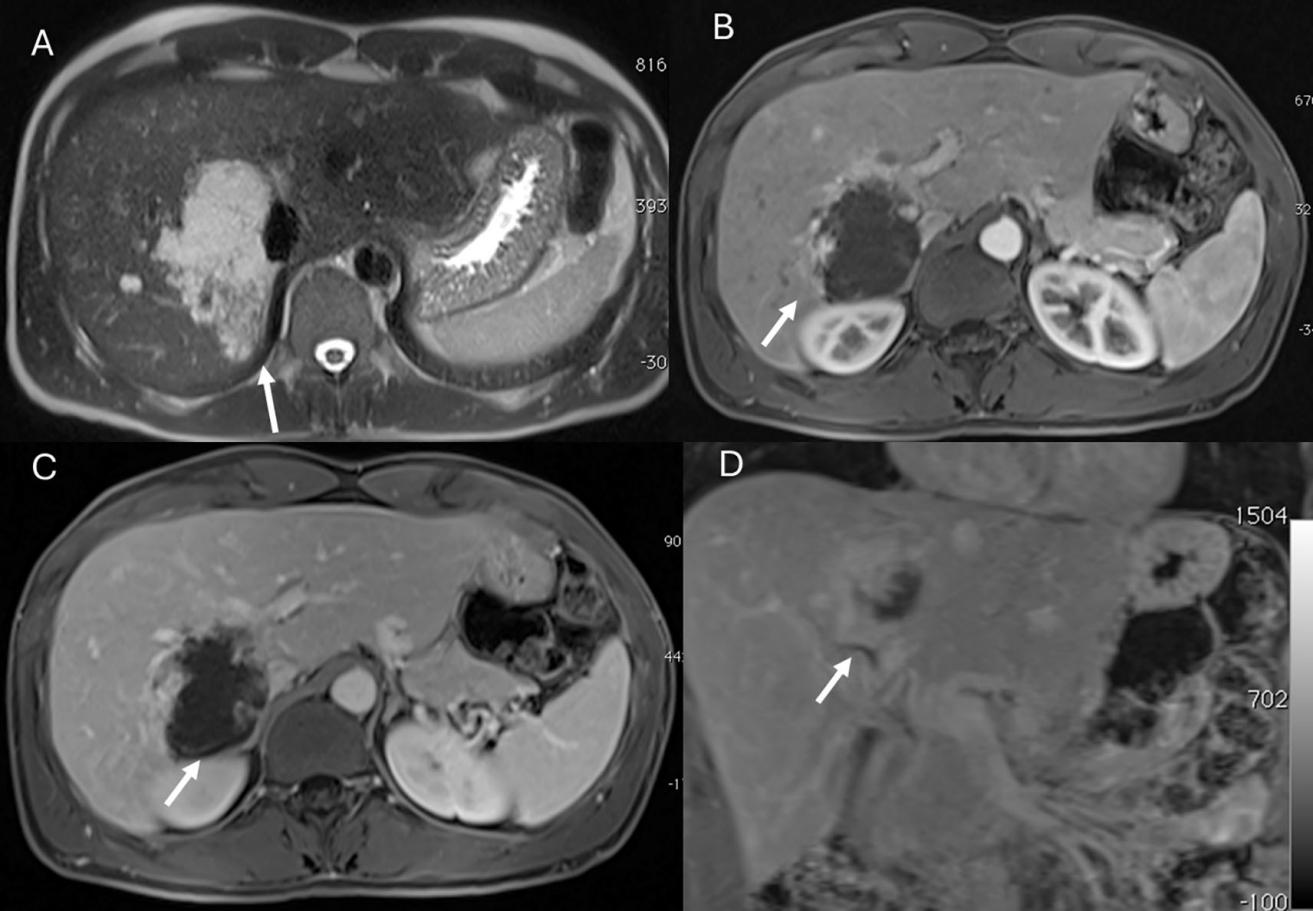
MRI assessment of liver hemangiomas (arrows). In **(A)** (T2-W sequence, axial plane), the hemangioma (arrow) shows hyperintense signal, and after non-specific contrast agent administration a progressive enhancement is seen **(B)** T1-W in axial plane during in arterial phase; **(C)** T1-W in axial plane during portal phase and **(D)** T1-W in coronal plane during late phase).

Incidental liver lesions are usually discovered during diagnostic evaluation performed for unrelated indications. Because the prevalence of benign lesions (**Figure 1**) is high, with at least one lesion identified in up to 15% of patients, exact characterization of incidentally detected lesions is a critical step in diagnostic management. Traditionally, MRI serves as a complementary diagnostic tool following initial assessment with more accessible and cost-effective modalities, such as ultrasound (US) or computed tomography (CT) (27). According to the American College of Radiology (ACR) Committee on Incidental Findings (28), the management of an incidental liver lesion depends on the patient’s risk category for having a malignant hepatic lesion. In particular, in low-risk patients—those with no history of malignancy, hepatic dysfunction, or hepatic risk factors—older patients (>40 years of age) are at higher risk than younger patients for malignancy (28). High-risk patients include those with known malignancies that commonly metastasize to the liver, cirrhosis, and/or other hepatic risk factors. Therefore, when evaluating a focal liver lesion, it is crucial to consider the patient’s clinical history. The ACR Committee identifies eight different clinical scenarios, each related to lesion size, presence of underlying liver disease, or history of oncological pathology (**Table 1**).

**Table 1:** Clinical scenarios and appropriate diagnostic tool, according to ACR.

Clinical scenario	Appropriate diagnostic tool
Initial US assessment; an indeterminate, greater than 1 cm liver lesion on normal liver without suspicion or evidence of extrahepatic malignancy or underlying liver disease	US with contrast agent Multiphase contrast CT study MRI with intravenous contrast medium
Indeterminate, greater than 1 cm liver lesion on initial imaging with CT (non-contrast or single-phase) or non-contrast MRI, in normal liver with no suspicion or evidence of extrahepatic malignancy or underlying liver disease	MRI without and with IV contrast Multiphase contrast CT study
Initial US assessment; an indeterminate, greater than 1 cm liver lesion in patient with known history of an extrahepatic malignancy	MRI without and with IV contrast Multiphase contrast CT study
Indeterminate, greater than 1 cm liver lesion on initial imaging with CT (non-contrast or single-phase) or non-contrast MRI. Known history of an extrahepatic malignancy	MRI without and with IV contrast Multiphase contrast CT study
Incidental liver lesion, greater than 1 cm on US, non-contrast or single-phase CT, or non-contrast MRI. Known chronic liver disease	US with contrast agent Multiphase contrast CT study MRI with intravenous contrast medium
An indeterminate, less than 1 cm liver lesion on US assessment, in patient with known history of an extrahepatic malignancy	MRI abdomen without and with IV contrast
Indeterminate, less than 1 cm liver lesion on initial imaging with CT (non-contrast or single- phase) or non-contrast MRI. Known history of an extrahepatic malignancy	MRI without and with IV contrast Multiphase contrast CT study
Incidental liver lesion, less than 1 cm on US, non-contrast or single-phase CT, or non-contrast MRI. Known chronic liver disease	MRI without and with IV contrast Multiphase contrast CT study

For scenario 1, in which a lesion is found on initial US assessment, an indeterminate liver lesion >1 cm in a normal liver without suspicion or evidence of extrahepatic malignancy or underlying liver disease, the second-level examination may include either contrast-enhanced US, multiphase CT, or MRI with intravenous (IV) contrast medium.

**Figure 2:**
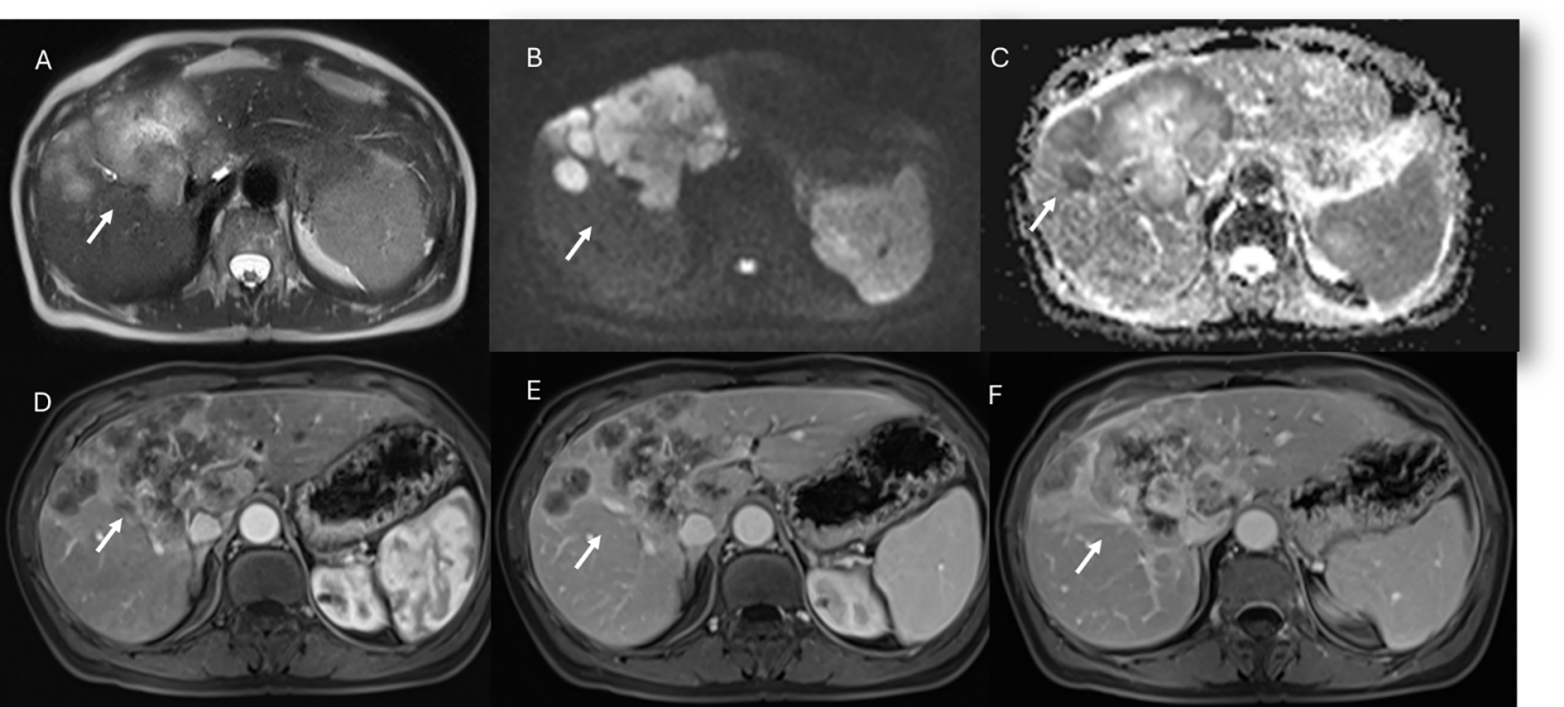
MRI assessment of non-mucinous colorectal liver metastases (arrows). In **(A)** (T2-W sequence, axial plane), the lesion (arrow) shows hyperintense signal with restricted diffusion on b = 880 s/mm² **(B)** and targetoid appearance on ADC map **(C)**. After non-specific contrast agent, rim enhancement is seen in the arterial phase **(D**, T1-W, axial**)**, with peripheral enhancement in the portal **(E)** and late phases **(F)** showing targetoid appearance from central necrosis.

**Figure 3:**
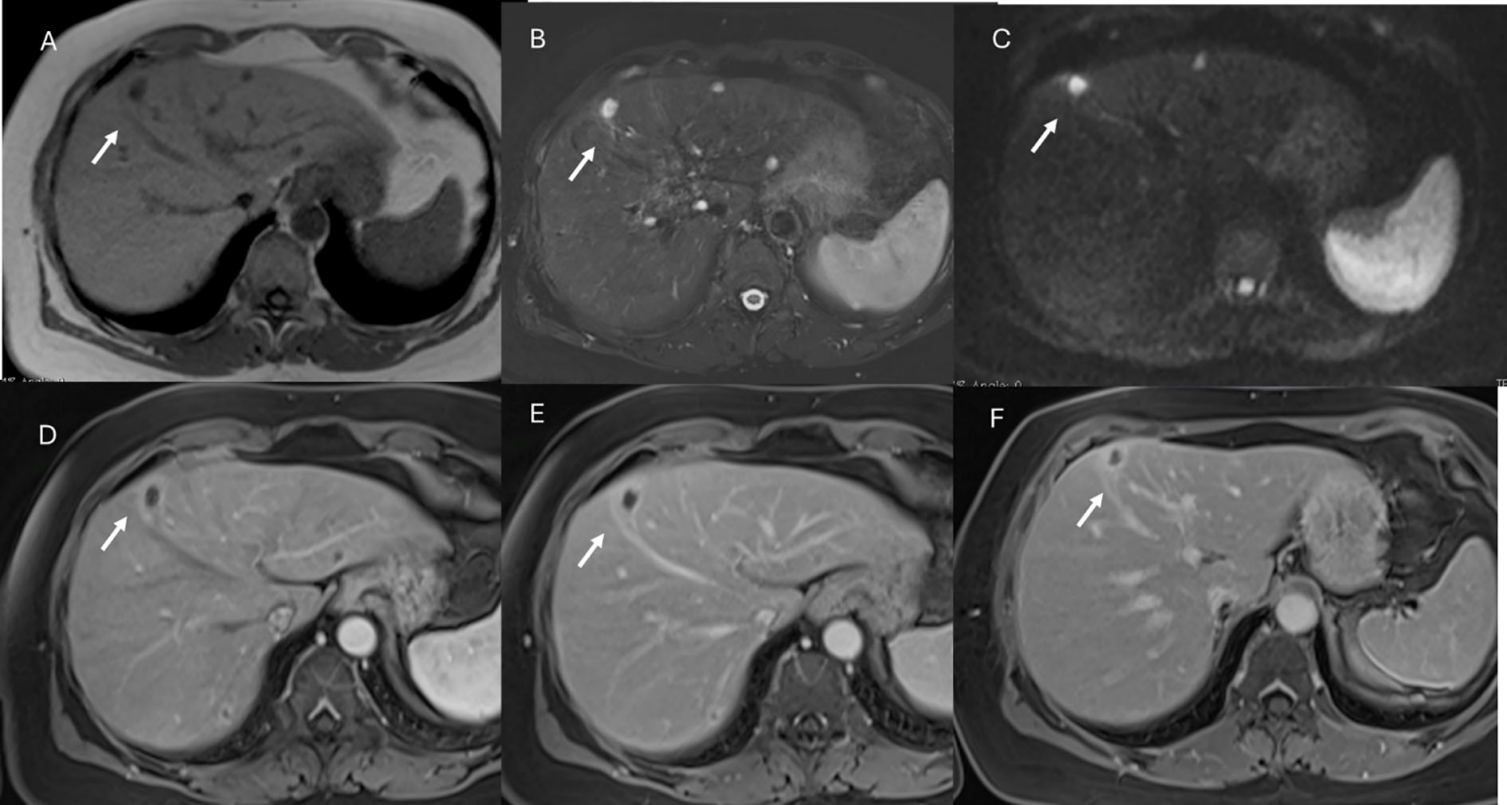
MRI assessment of mucinous colorectal liver metastases (arrows). In **(A)** (T1-W sequence, axial plane), the lesion (arrow) shows hypointense signal, with very high signal on T2-W FS sequence **(B)** and restricted diffusion on b = 880 s/mm² **(C)**. After non-specific contrast agent, rim enhancement is seen in the arterial phase **(D**, T1-W, axial**)**, with progressive enhancement in the portal **(E)** and late phases **(F)**.

For scenario 2, characterized by an indeterminate liver lesion >1 cm on initial imaging with CT (non-contrast or single-phase) or non-contrast MRI, in a normal liver with no suspicion or evidence of extrahepatic malignancy or underlying liver disease, both MRI without and with IV contrast and multiphase contrast-enhanced CT are considered appropriate. The suggested scenario 2 diagnostic management also applies to scenario 3, in which an indeterminate liver lesion >1 cm is found on initial US assessment in a patient with a known history of extrahepatic malignancy (**Figures 2** and **3**), as well as to scenario 4 (indeterminate liver lesion >1 cm on initial imaging with CT [non-contrast or single-phase] or non-contrast MRI in a patient with a known history of extrahepatic malignancy).

Regarding scenario 5 (incidental liver lesion >1 cm on US, non-contrast or single-phase CT, or non-contrast MRI, in a patient with known chronic liver disease), in addition to MRI without and with IV contrast, multiphase contrast-enhanced CT or contrast-enhanced US may also be appropriate.

For scenario 6, characterized by an indeterminate liver lesion <1 cm on US assessment in a patient with a known history of extrahepatic malignancy, only abdominal MRI without and with IV contrast is considered appropriate. In contrast, for scenario 7 (indeterminate liver lesion <1 cm on initial imaging with CT [non-contrast or single-phase] or non-contrast MRI in a patient with a known history of extrahepatic malignancy) and scenario 8 (incidental liver lesion <1 cm on US, non-contrast or single-phase CT, or non-contrast MRI in a patient with known chronic liver disease), either abdominal MRI without and with IV contrast or abdominal CT with multiphase IV contrast are appropriate.

It is clear that the choice of diagnostic tool will depend on operator experience, availability of technology, and patient-specific characteristics. Likewise, the choice of MRI contrast agent will depend strictly on the clinical question. However, in a low-risk patient, an incidental hepatic lesion <1 cm without suspicious features does not require further workup, whereas in the presence of suspicious features, MRI should be considered. An incidental hepatic lesion ≥1 cm with suspicious features requires further workup with MRI or biopsy, depending on lesion size, imaging characteristics, and the patient’s risk level (28).

### HCC patients

Patients with HCC or at risk for HCC deserve separate consideration (**Table 2**).

**Table 2:** HCC patients: clinical scenarios and appropriate diagnostic tool, according to ACR.

Clinical scenario	Appropriate diagnostic tool
Screening	US; poor US visualization, NC-AMRI
Diagnosis	US with contrast agent Multiphase contrast CT study MRI with intravenous contrast medium
Staging	MRI abdomen without and with IV contrast; MRCP sequence may be appropriate if there is concern for biliary involvement
Treatment assessment and surveillance	MRI abdomen without and with IV contrast CT abdomen with IV contrast multiphase study

With regard to surveillance, the AASLD currently recommends US, with serum AFP, every 6 months (29). Similarly, the European Association for the Study of the Liver (EASL) guidelines also recommend surveillance with US every 6 months (30). However, several studies have suggested a potential role for MRI in the screening setting (31–33). The ACR further suggests that MRI may be an option for patients with poor visualization on US screening examinations, such as those with non-alcoholic fatty liver disease (NAFLD) or non-alcoholic steatohepatitis (NASH). Nevertheless, its feasibility in clinical practice is limited due to lower scanner availability and higher cost.

A recent meta-analysis (34), which included 27 studies (2009–2023) from Western (n = 14) and Eastern (n = 13) countries, evaluated the diagnostic performance of non-contrast abbreviated MRI (NC-aMRI) compared to contrast-enhanced abbreviated MRI (CE-aMRI) for HCC surveillance. NC-aMRI, reported in 21 studies, demonstrated 83% (79–87%, 63%) sensitivity and 91% (88–93%, 67%) specificity. CE-aMRI, reported in 15 studies, demonstrated 88% (84–91%, 64%) sensitivity and 94% (90–96%, 78%) specificity, with no statistically significant differences in sensitivity (p = 0.078) or specificity (p = 0.157). Subgroup analysis in NC-aMRI studies showed significant differences in sensitivity in high-prevalence chronic hepatitis B (87% vs. 78%, p = 0.003) and in studies from Eastern countries (86% vs. 76%, p = 0.018). Specificity was significantly higher in chronic hepatitis C (94% vs. 90%, p = 0.009). Meta-regression identified study heterogeneity sources as the inclusion of patients with chronic hepatitis B (p = 0.008) and the geographic region of the study (p = 0.030) (34).

A retrospective study (35) of 1,853 Child-Pugh class A or B adults with chronic hepatitis B or cirrhosis who underwent NC-aMRI (December 2018–August 2022) reported effective HCC surveillance, particularly for early and very early-stage disease. Detection rates for early and very early-stage HCC were 95.1% (58/61, 72.2–100.0) and 70.5% (43/61, 51.0–95.0), respectively. Among 375 patients with inadequate prior US, early- and very early-stage detection rates were 94.7% (18/19, 56.2–100.0) and 57.9% (11/19, 28.9–100.0) (35).

Several studies have highlighted that US is operator-dependent and has poor performance in patients with obesity or NASH (9, 36, 37), which supports the role of MRI as a valid diagnostic tool in these populations (27, 38).

The recommended diagnostic tools for HCC are contrast-enhanced US (CEUS), multiphase CT, multiphase MRI with extracellular contrast agents (ECA), and multiphase MRI with hepatobiliary agents (HBA) (18). Several studies suggest that MRI offers higher sensitivity with similar specificity compared to CEUS or multiphase CT (18). However, the choice of diagnostic tool must be individualized, considering patient factors such as breath-holding capability, claustrophobia, body habitus, renal function, and comorbidities (e.g., allergies). Institutional factors, including technology availability and expertise, also play a role (18).

Non-invasive diagnosis of HCC should be based on LI-RADS CT/MR v2018 or LI-RADS CEUS v2017 criteria. For CT/MRI, the major imaging features combined to reach a diagnosis include tumor size, rim and non-rim arterial hyperenhancement, and peripheral or non-peripheral washout. For CEUS, non-rim arterial hyperenhancement with late-onset (>60 s) and mild washout are combined to establish the diagnosis (39).

**Figure 4:**
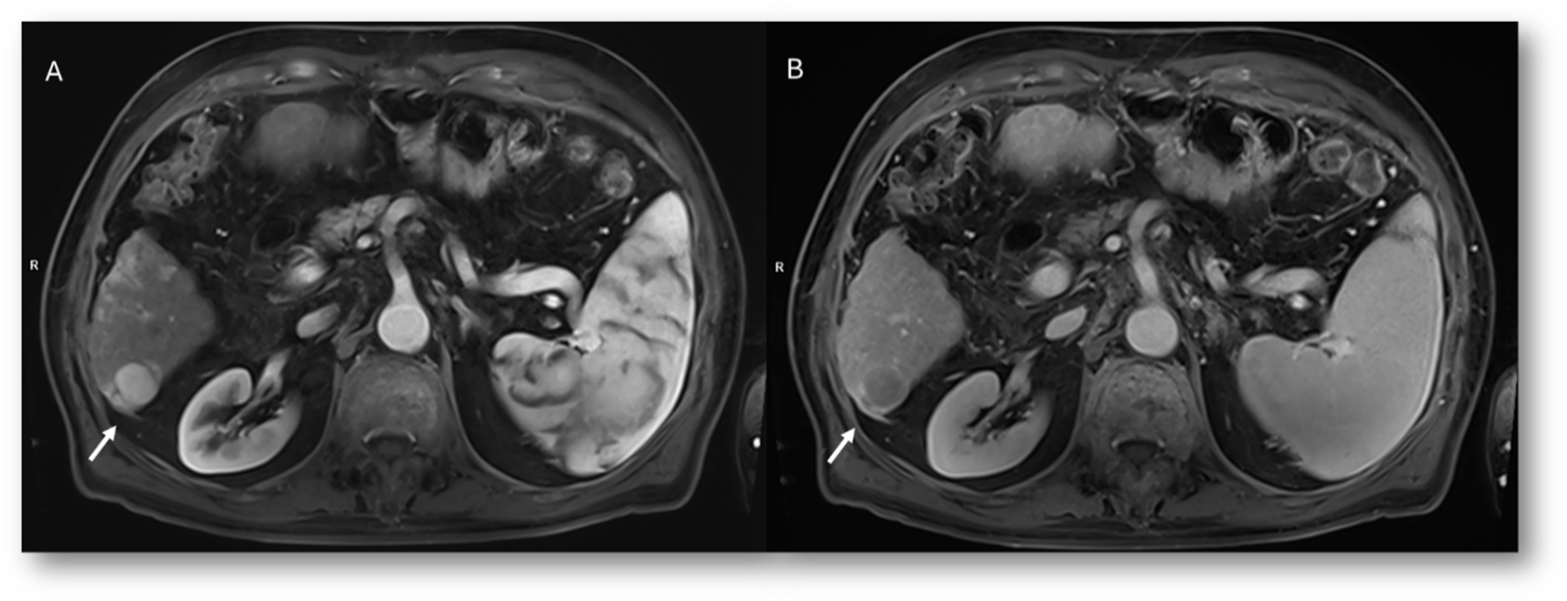
MRI assessment of HCC (arrows). Contrast evaluation with non-specific contrast agent. In **(A)** and **(B)** (T1-W FS sequences, axial plane), the lesion shows APHE during arterial phase **(A)** and washout in portal phase **(B)**, with capsule appearance. These are typical major features according to LI-RADS v2018.

According to EASL guidelines (39), multiphasic CT or dynamic contrast-enhanced MRI should be considered, without preference, though extracellular contrast agents are recommended over gadoxetic acid (**Figure 4**). CT, however, is preferred over MRI for staging of distant disease.

For staging, the liver containing at least one HCC should be assessed with an imaging technique that provides complete anatomic coverage. Multidetector CT (MDCT) and MRI are the only acceptable techniques for local tumor staging. Although CT is sensitive for detecting primary tumors (71–87%), its sensitivity for additional lesions is lower. Therefore, MRI should be preferred in multifocal HCC, particularly for lesions <2 cm.

**Figure 5:**
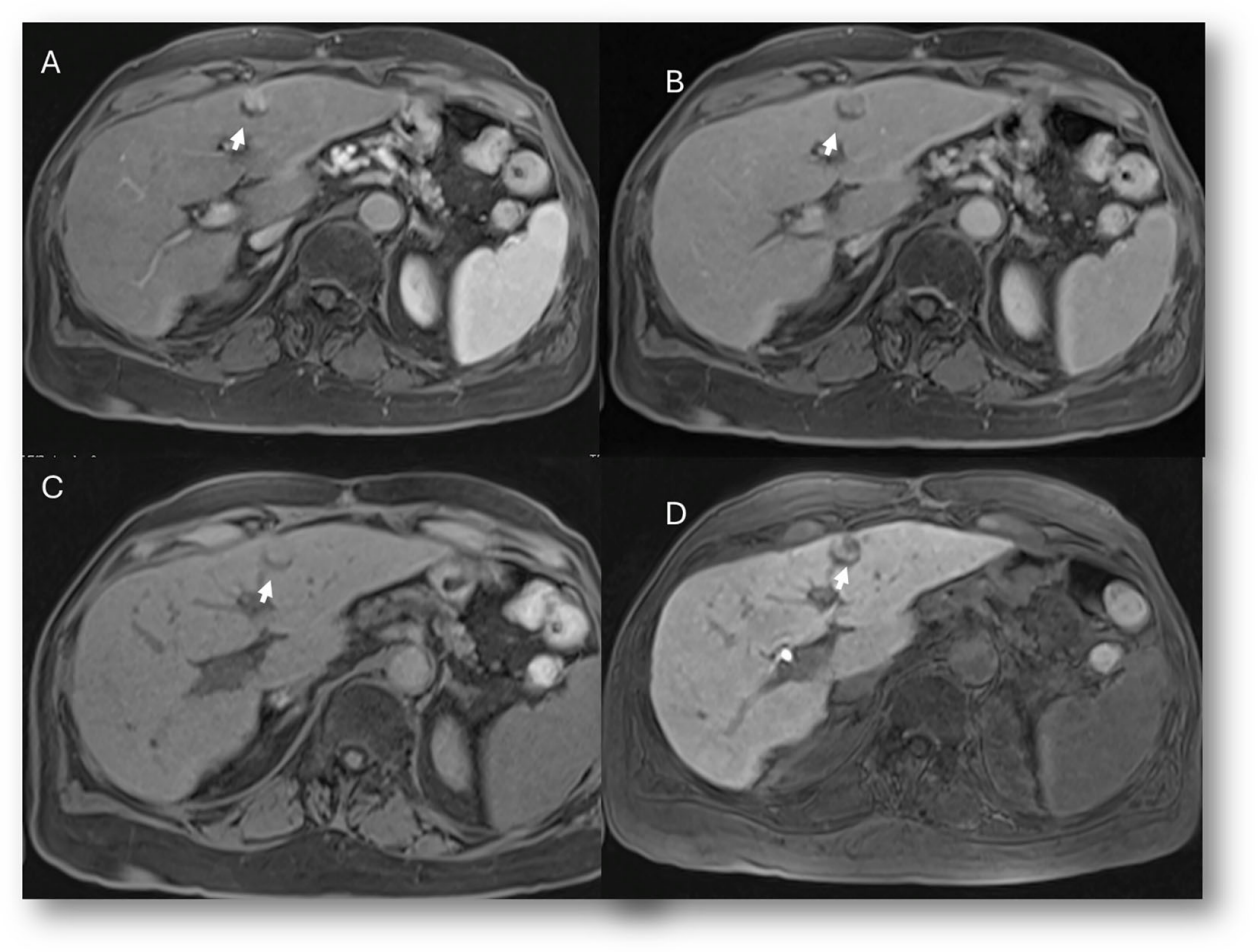
MRI assessment of nodule-in-nodule in early HCC (arrows). Contrast study with specific agent (Gd-EOB-DTPA). In **(A)** (T1-W FS sequence, axial plane), the lesion shows APHE during arterial phase, while during portal **(B)** and transitional phases no washout is present. **(C)** Only the nodule-in-nodule shows hypointense signal in the hepatospecific phase **(D)**.

MRI with ECA is highly sensitive for additional lesions >20 mm (100%) and 10–20 mm (84%), but less sensitive for lesions <10 mm (32%). The addition of MRI with gadoxetate can change staging in 14–28% of patients, influencing management in 13–19%. Detection of additional HCC on gadoxetate-enhanced MRI (**Figure 5**) following MDCT reduces HCC recurrence rates by 28% and overall mortality by 35% (18). Thus, abdominal MRI without and with IV contrast allows comprehensive assessment of the primary lesion and vascular involvement. Inclusion of MR cholangiopancreatography (MRCP) may be appropriate if biliary involvement is suspected.

Imaging is also necessary after liver-directed therapy to assess treatment response. The goal is to identify treatment success, complications, or viable tumor that may require retreatment (18). In HCC patients undergoing ablation or systemic therapy, abdominal MRI without and with IV contrast or multiphase contrast-enhanced CT are recommended for treatment response evaluation and for surveillance after complete response (27, 39–42). For post-treatment complications, MRI with HBA is the only diagnostic tool that can identify a biliary fistula, demonstrated by leakage of contrast medium from a bile duct (43, 44). Additionally, functional MRI data (e.g., T1 maps, fat fraction maps) may help assess residual liver function.

In summary, MRI is a valuable tool in various clinical scenarios—screening, detection, staging, and treatment evaluation. However, beginners must recognize that the choice of diagnostic modality depends on patient characteristics (e.g., ability to cooperate) and technology availability. Therefore, adequate knowledge of multiparametric MRI sequences and contrast agents is essential.

### Study protocol

MRI is probably the most operator-dependent imaging technique, and this is closely related to the radiologist’s preference for the individual sequences acquired. This is also linked to continuously evolving technology, with vendors providing different options for specific sequences—for example, modern Dixon techniques compared to more traditional post-contrast sequences.

Liver MRI is a well-established multiparametric imaging modality. A standard protocol should include T2-weighted (T2W) sequences, T2W sequences with fat suppression (FS), and in-phase/opposed-phase (IP/OP) gradient-echo (GRE) T1 sequences (45). These conventional, morphological sequences allow detection and characterization of liver lesions, although diagnostic performance also depends on lesion size and hepatic parenchymal condition (46–48). Typically, a markedly hyperintense lesion on T2W images (signal intensity similar to the gallbladder) with hypointense signal on T1W sequences represents a simple cyst. By contrast, a suspicious lesion may appear hyperintense on T2W images but with less intensity than the gallbladder and hypointense on T1W images (49–51).

Single-shot fast spin echo (SSFSE) images are usually acquired at the beginning of the liver MRI protocol. Several authors recommend heavily T2W images, with an echo time (TE) >160 ms, ideally 180–200 ms. These longer TEs can help differentiate cysts and hemangiomas from solid liver tumors (46–48). This sequence involves a single excitation pulse followed by a long train of 180° refocusing pulses. It can be further accelerated using half-Fourier acquisition (HASTE). Because these sequences acquire each slice in ≤1 s and cover the liver in one or two breath-holds, they are more resistant to susceptibility and motion artifacts.

Fast/turbo spin echo (FSE/TSE) T2W sequences are generally used, with a repetition time (TR) of ~2,500 ms and a TE of 60–120 ms (ideally 80–100 ms), producing moderate T2 weighting (45). Since FSE T2W sequences are affected by magnetization transfer effects that preserve high fat signal intensity, fat suppression should be routinely applied. These sequences primarily depict fluid content, aiding distinction between solid and cystic-like focal lesions. The spleen should be used as an internal reference, since most malignant lesions show signal intensity similar to that of the spleen.

T1W sequences detect fat and other substances with high T1 signal, such as hemorrhage, proteinaceous material, copper, or glycogen. GRE sequences are generally used. Because they are highly sensitive to susceptibility artifacts, they are useful for detecting iron, calcium, air, or metal. Currently, dual-echo sequences are employed to generate in-phase and opposed-phase images, exploiting cancellation effects of coexisting fat/water molecules within the same voxel. To minimize T2* decay, the TE should be as short as possible. The out-of-phase TE must be shorter than the in-phase TE (usually 2.3 ms vs. 4.6 ms at 1.5T) (45). These sequences allow detection of intracellular fat in both lesions and liver parenchyma. In out-of-phase images, interfaces between tissues of different resonance frequencies (e.g., pure fat and water) appear dark, creating the so-called “India ink” artifact. The primary application of IP/OP sequences is the identification of microscopic fat content within a lesion, demonstrated by signal drop on opposed-phase compared to in-phase images. This sequence is helpful in diagnosing fatty liver, focal fatty sparing/infiltration, HCC, and hemochromatosis (52).

3D sequences, often based on modified Dixon techniques, are increasingly used. Dixon sequences allow accurate fat–water separation, enabling precise assessment of hepatic steatosis and lesion characterization. They are especially valuable in evaluating metabolic liver disorders and ensuring comparability of liver fat quantification across MRI platforms. With Dixon, hepatic steatosis is quantified as the proportion of fat relative to water, providing a reliable biomarker for tracking liver fat in conditions such as NAFLD. Proton density fat fraction (PDFF) is precise, reproducible, and well suited for monitoring liver fat, including chemotherapy-associated steatosis (CASH) (53). Dixon can also be performed after contrast administration. Advantages include improved spatial resolution, shorter acquisition times, and additional diagnostically useful sequences. Limitations include potentially lower signal-to-noise ratio with thinner sections and fat–water swap artifacts (54, 55).

3D GRE T1W FS sequences (e.g., LAVA, VIBE, THRIVE) are typically used for dynamic contrast-enhanced imaging.

MR cholangiopancreatography (MRCP) is an optional sequence when biliary tract involvement is suspected, although image quality may be degraded in cases of significant ascites (56).

Nowadays, diffusion-weighted imaging (DWI) is considered mandatory, especially for detecting very small lesions. However, diffusion restriction does not necessarily indicate malignancy, since even benign lesions (e.g., hepatic hemangiomas) may demonstrate this feature (57–60). DWI evaluation can be qualitative (persistence of hyperintense signal with progressively higher b values) or quantitative (assessment of the apparent diffusion coefficient [ADC]).

Beyond conventional DWI, more sophisticated approaches are available to analyze water molecular motion: intravoxel incoherent motion (IVIM) and diffusion kurtosis imaging (DKI). IVIM is a biexponential model that separates tissue diffusivity and tissue perfusion (61). DKI assumes that water molecules diffuse within a voxel according to a non-Gaussian model. It evaluates the kurtosis coefficient (K), which reflects deviation from Gaussian diffusion, and the diffusion coefficient (D), corrected for non-Gaussian bias. DKI is thought to be more sensitive to microstructural complexity than standard DWI (62). However, while conventional DWI is an integral part of the liver study protocol, IVIM and DKI remain experimental. Their limitations include increased acquisition time (approximately 50% longer) and the requirement for specialized tools to evaluate the resulting parametric maps, which are not always available on reporting workstations.

Echo planar imaging (EPI) sequences are widely used for DWI. These are essentially T2W single-shot images with fat suppression, which rapidly capture the diffusion signal before it decays, while remaining relatively insensitive to patient motion (62). The repetition time (TR) should exceed 2,500 ms, at least three times the T1 of a typical metastasis, to minimize T1 saturation and improve ADC accuracy. Image quality deteriorates as TE increases, so TE should be minimized, often by reducing the acquisition matrix to around 128 × 128 (62).

Different DWI series are acquired by varying gradient strength and duration, referred to as the b value.

- One series should be obtained with a b value of 0, meaning no diffusion weighting. This sequence yields information similar to that of T2W FS images.
- A low b value (<100) is recommended for lesion detection. These images produce a “black blood” effect, improving conspicuity of lesions adjacent to vessels.

High b values (e.g., b = 800) are important for lesion characterization, offering greater SNR and CNR and being less affected by artifacts (62).

Contrast-enhanced MRI remains the cornerstone of liver MRI, particularly for lesion categorization (63–65). Although extracellular agents are the most widely used contrast media, hepatobiliary agents can provide functional data and increase diagnostic value. The choice of contrast medium is the responsibility of the radiologist, who must consider the clinical question, agent properties, and patient-specific factors such as renal function and bilirubin levels (66).

Another critical point is the correct timing of post-contrast acquisitions, which must be adapted to both the lesion type (e.g., hypervascular lesions such as HCC) and patient factors (e.g., cardiac function). Automated bolus-tracking systems are recommended, triggering acquisition when a predefined signal intensity threshold is reached.

Currently, vendors provide various T1W sequences for contrast imaging. These differ in sequence development, but each radiologist will choose the sequence deemed most appropriate for the clinical purpose. Some sequences also provide parametric maps for liver function assessment.

Three-dimensional FS GRE T1W sequences are the backbone of dynamic contrast-enhanced MRI, performed before and across successive phases after IV contrast administration. These sequences have sufficient temporal resolution for single breath-hold acquisition, with good spatial resolution and SNR. Parallel imaging can either increase spatial resolution or shorten acquisition time. TR and TE should be kept as short as possible: a short TR reduces acquisition time and increases T1 weighting, while a short TE minimizes susceptibility artifacts. Typical flip angles range from 10° to 15°. Fat suppression is essential to improve lesion visualization and reduce abdominal wall motion artifacts.

The ability to extract liver function data is particularly valuable in presurgical assessment, since the major limiting factor for extensive resections is the volume of residual functional liver (67).

Other sequences not yet integral to standard protocols, but of growing interest due to their functional value, include MR elastography (MRE), T1 mapping, and R2/T2 imaging (67).

Despite all developments, MR diagnostic performance is still affected by artifacts, especially those caused by motion. Measures can be taken to avoid voluntary movements, but physiologic motion is inherent to any liver imaging protocol, whether it results from breathing or cardiac motion, blood flow and vessel pulsation, or even gastrointestinal peristalsis. Movement during image acquisition leads to blurring and ghosting, image duplicates from misplaced signal that may hamper image interpretation. Strategies to reduce motion artifacts include signal averaging, ultrafast motion-resistant sequences, and ubiquitous use of fat suppression. Successful MRI requires significant patient cooperation to obtain diagnostic-quality images. This requires the patient to remain still for the entire examination to minimize motion artifact and often to follow breath-holding instructions for liver imaging. There are several methods for mitigating patient motion specific to MRI technique. These methods can be grouped into two distinct strategies: 1) increasing the speed of image acquisition to reduce the effects of motion or to conclude the exam before a calm or asleep patient rouses, and 2) acquiring or reconstructing the MR signal in such a way as to account for and minimize that motion when it occurs. There are many imaging techniques that dramatically increase the speed of image acquisition. These techniques include, but are not limited to, partial Fourier acquisition of k-space, simultaneous multislice imaging, parallel imaging, fast/turbo spin echo, keyhole k-space sampling (a subtype of partial Fourier acquisition primarily used in time-resolved angiography), and compressed sensing; these techniques can often be used in conjunction to further increase imaging speed. For example, the Siemens HASTE (Half-Fourier Acquisition Single-shot Turbo Spin Echo) combines echo-planar fast spin echo imaging with partial Fourier (in this case, approximately half) k-space sampling, yielding images that within individual slices have little motion degradation/artifact.

Breath-hold, free breathing, and respiratory-triggering techniques are used to control breathing motion, the last two leading to an increase in scan time. The use of navigator echoes for respiratory triggering is among the most popular and well-succeeded techniques.

Advancements in motion monitoring and motion correction opened the door to free-breathing liver T1 dynamic acquisition. Liver motion was monitored by external devices or navigator pulse sequence for years; however, recent advanced radial k-space acquisition methods can detect motion information from acquired k-space data itself. These advanced self-navigation radial pulse sequences allow us to acquire free-breathing T1 dynamic MR imaging. The introduction of compressed-sensing (CS) accelerated sequences, which allow more flexible k-space sampling, has paved the way for new strategies of reducing motion artifacts. Golden-angle radial sparse parallel MRI (GRASP) combines the CS reconstruction with a motion-insensitive radial k-space sampling using an efficient golden-angle trajectory. As a result, DCE of the upper abdominal organs in free breathing is possible with good image quality in various organs. The disadvantages of GRASP include its limited availability on older MR systems and the possibility of long reconstruction times. In fact, the radial readout technique is generally more time-consuming than the Cartesian readout technique. Hence, the sequence is not suitable for fast dynamic imaging because of its low temporal resolution.

Cardiac motion affects mostly the left liver lobe and can be overcome by ECG or pulse-triggering techniques, but again with an acquisition time penalty.

The standard use of multichannel, multielement phased-array coils has allowed the use of parallel imaging technique, which has dramatically improved SNR, accelerating the k-space acquisition, reducing scan times and susceptibility artifacts. Acceleration factors, or the number of lines of k-space acquired in parallel, are typically limited by the development of residual artifacts and severe signal loss; thus, a factor higher than 2 is rarely used.

Since its clinical implementation, DWI-MRI has been used largely as a tool to complement conventional MRI sequences. However, diffusion-weighted MRI was associated with relatively low spatial resolution that hindered anatomic assessment of these images. Recent advances in imaging equipment hardware and post-processing techniques have helped improve the image quality of DW-MR images. In addition, the application of artificial intelligence (AI) is an emerging technical development area for liver MR imaging. It has been applied to motion artifact reduction, contrast bolus detection, scan prescription automation, and image recognition to select the appropriate dynamic phase. Recently, deep learning (DL) approaches showed great potential for MR image denoising and image-quality–degrading artifact correction. Several authors have demonstrated significant improvements in image-analysis tasks using DL-based convolutional neural network techniques. The promising capabilities and performance of DL techniques in various problem-solving domains have motivated researchers to adapt DL methods to medical image analysis and quality enhancement tasks.

### Abbreviated protocol

The abbreviated protocols deserve a special mention.

As previously mentioned, several authors are evaluating the efficacy of different protocols in HCC screening (32–35), and in the literature it is possible to find data on the use of abbreviated protocols also in the setting of liver metastases (68–75). The concept of an abbreviated protocol is based on the idea that, in less examination time, it is possible to acquire all the information necessary for the management of a patient using only part of the sequences that normally fall within a standardized study protocol (76). The advantages are numerous: less discomfort for the patient, due to shorter examination times; economic savings linked to reduced use of resources (for example, contrast agents); improved sustainability in radiology with minimal social impact; and the possibility of examining more patients, thereby reducing waiting lists. In the literature the findings are discordant. There are examples of abbreviated protocols that also use contrast medium (75, 77, 78) and, in some cases, hepatospecific agents. In such situations, it would not seem suitable to omit acquisition of all study phases, given the time necessary for the biliary excretion phase. It is therefore clear that an abbreviated protocol is acceptable only if it does not cause a loss of information necessary for diagnosis. Thus, in the context of liver pathology, such diagnostic management must remain subordinate to the clinical question. If we consider abbreviated protocols without the use of contrast, it is clear that these fall outside a characterization setting. In fact, it is well established that contrast medium is fundamental in the characterization process of a lesion (79, 80), and dynamic contrast studies are necessary to evaluate HCC response to treatment (81–83). The use of an abbreviated protocol seems useful (**Table 3**) in the detection of lesions—particularly in screening, surveillance of lesions with already known nature, evaluation of response to conventional chemotherapeutic drugs (which are responsible only for a reduction in size), and in the pre-surgical phase after treatment of liver metastases. However, in this context, liver-specific contrast medium may also be useful for functional evaluation of the residual parenchyma in patients scheduled for major liver surgery (84, 85).

**Table 3:** Appropriateness for non-contrast abbreviated protocol.

Clinical question	Appropriateness for non-contrast abbreviated protocol
HCC screening	Yes
Lesion detection	Yes
Lesion characterization	No
Staging	No
Response assessment after conventional chemotherapy	Yes
Response assessment after ablation, radiotherapy and target therapy	No
Pre-surgical setting	Only if there is no need for a functional evaluation of the liver parenchyma or vascular assessment

An abbreviated protocol without contrast medium or dynamic study would not seem applicable (**Table 3**) when there is a need to characterize a lesion, to evaluate response to targeted therapies, radiotherapy, or ablative treatments, to stage lesions for proper vascular assessment, or to conduct pre-transplant liver evaluation (86–89). Nevertheless, there is an evident need for new and more robust scientific evidence to support this thesis.

In **Table 4**, examples of standard and abbreviated protocols are reported.

**Table 4:** Examples of standard and abbreviated MR sequence parameter protocols for liver metastases detection in our institution at 1.5 T.

Sequence	Orientation	TR/TE/FA (ms/ms/deg.)	AT (min)	Acquisition matrix	ST/gap (mm)	FS
Standard protocol						
Trufisp T2-W	Coronal	4.30/2.15/80	0.46	512 × 512	4/0	without
HASTE T2-W	Axial	1500/90/170	0.36	320 × 320	5/0	without and with (SPAIR)
HASTE T2W	Coronal	1500/92/170	0.38	320 × 320	5/0	without
SPACE T2-W	Axial	4471/259/120	4.2	384x450	3/0	with
In-Out phase T1-W	Axial	160/2.35/70	0.33	256 × 192	5/0	without
VIBE T1-W	Axial	4.80/1.76/30	0.18	320 × 260	3/0	with (SPAIR)
DWI	Axial	7500/91/90	7 (for seven b values)	320×260	3/0	with (SPAIR)
Abbreviated Protocol	(without contrast agents)					
SPACE T2-W	Axial	4471/259/120	4.2	384x450	3/0	with
DWI	Axial	7500/91/90	7 (for seven b values)	320×260	3/0	With (SPAIR)

W, weighted; TR, repetition time; TE, echo time; FA, flip angle; AT, acquisition time; SPAIR, spectral adiabatic inversion recovery; VIBE, volumetric interpolated breath hold examination; HASTE, half-Fourier-acquired single-shot turbo spin echo.

### Contrast agents

Contrast MR agents play a critical role during the detection and characterization phases of focal or diffuse hepatic disease, improving detection rates by increasing lesion-to-liver contrast and, according to their pharmacokinetics, enabling characterization by showing changes in vascular, extracellular, or intracellular volumes, as well as modifications in transfer rates between these compartments (90). These data and transfer rates can be quantified during post-contrast studies, offering biomarkers for the assessment of liver diseases (90). For liver MRI, contrast agents can be classified as non-specific agents, which distribute into vascular and extracellular extravascular spaces (as in CT studies), and specific agents, which are taken up by hepatic cells—either by Kupffer cells or hepatocytes.

**Figure 6:**
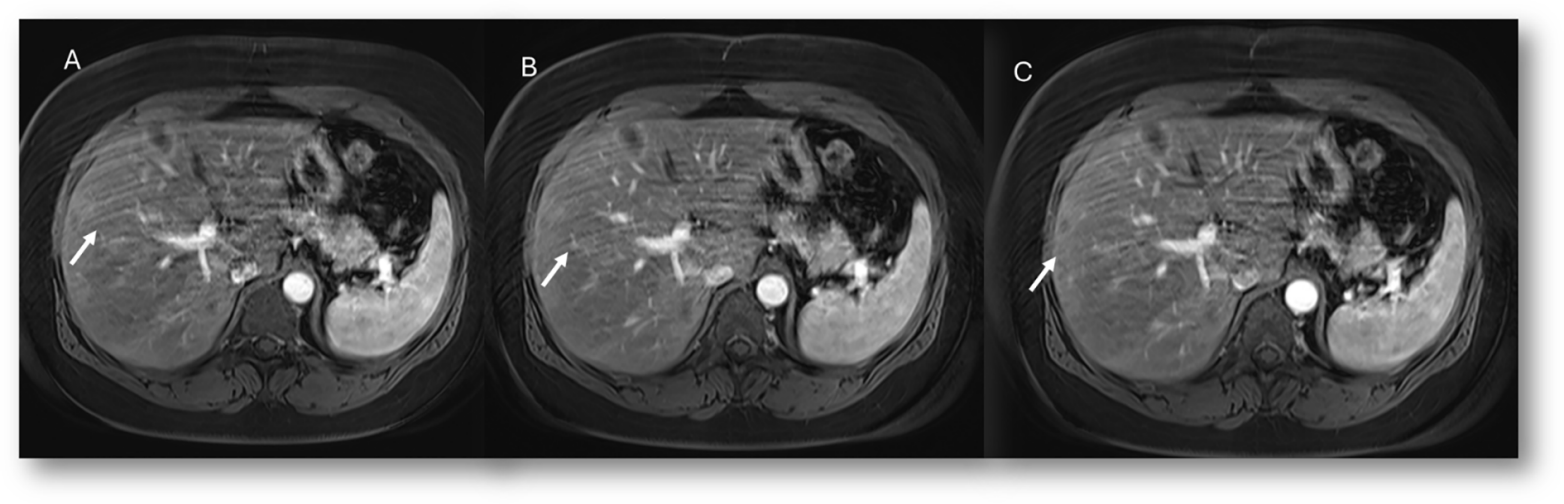
MRI assessment of colorectal liver metastases with Gd-EOB-DTPA contrast during multi-arterial phase: motion artifacts on all phases with low image quality. **(A–C)** Sequence acquired on 3T VIDA System (Siemens, Germany) with deep learning AI technology.

Currently, the most widely used specific agents are those taken up by hepatocytes. These include two types: gadolinium ethoxybenzyl dimeglumine, also known as gadoxetate dimeglumine (Gd-EOB-DTPA; marketed as Primovist in Europe and Eovist in the US, Bayer HealthCare), and gadobenate dimeglumine (Gd-BOPTA; MultiHance, Bracco, Milan, Italy). Although both can be injected as intravenous boluses, they show different contrast kinetics. Gd-BOPTA behaves similarly to non-specific contrast media and allows better vascular assessment. By contrast, studies with Gd-EOB-DTPA are characterized by early uptake by hepatocytes, beginning at about 5 min. Consequently, there is no true late phase; instead, a transition phase is observed (90). In addition, the quality of the arterial phase can be compromised by motion artifacts (**Figure 6**)—an important factor to keep in mind when selecting contrast for studies requiring high-quality arterial imaging. However, the introduction of artificial intelligence algorithms appears to improve arterial phase quality in Gd-EOB-DTPA studies (91–94).

With regard to the hepatobiliary phase, two factors are important: acquisition time and excretion rate.

The hepatobiliary phase occurs at 20 min with Gd-EOB-DTPA, while with Gd-BOPTA it is performed at 60–120 min. An examination with Gd-BOPTA therefore requires longer scanner time, though this can be mitigated by allowing the patient to leave the magnet room and return for the final acquisition. By contrast, Gd-EOB-DTPA studies follow the same duration as a standard protocol, with the contrast-enhanced sequences acquired first, followed by T2W sequences, DWI, and finally the liver-specific phase. It should also be remembered that cholangiographic sequences must be acquired before administration of liver-specific contrast. Alternatively, they can be obtained after a non-specific contrast agent for better-quality information, since gadolinium-based contrast produces a T2 effect.

With regard to the excretion rate in the liver-specific phase, with Gd-EOB-DTPA, approximately 50% of the administered dose in the normal human liver is transported through the hepatocytes and excreted into the bile, a proportion much higher than that of Gd-BOPTA, which has only up to 5% hepatobiliary excretion. This involves a consideration of liver function, which in turn can influence this rate; therefore, it seems appropriate to know the bilirubin values before choosing the contrast.

**Figure 7:**
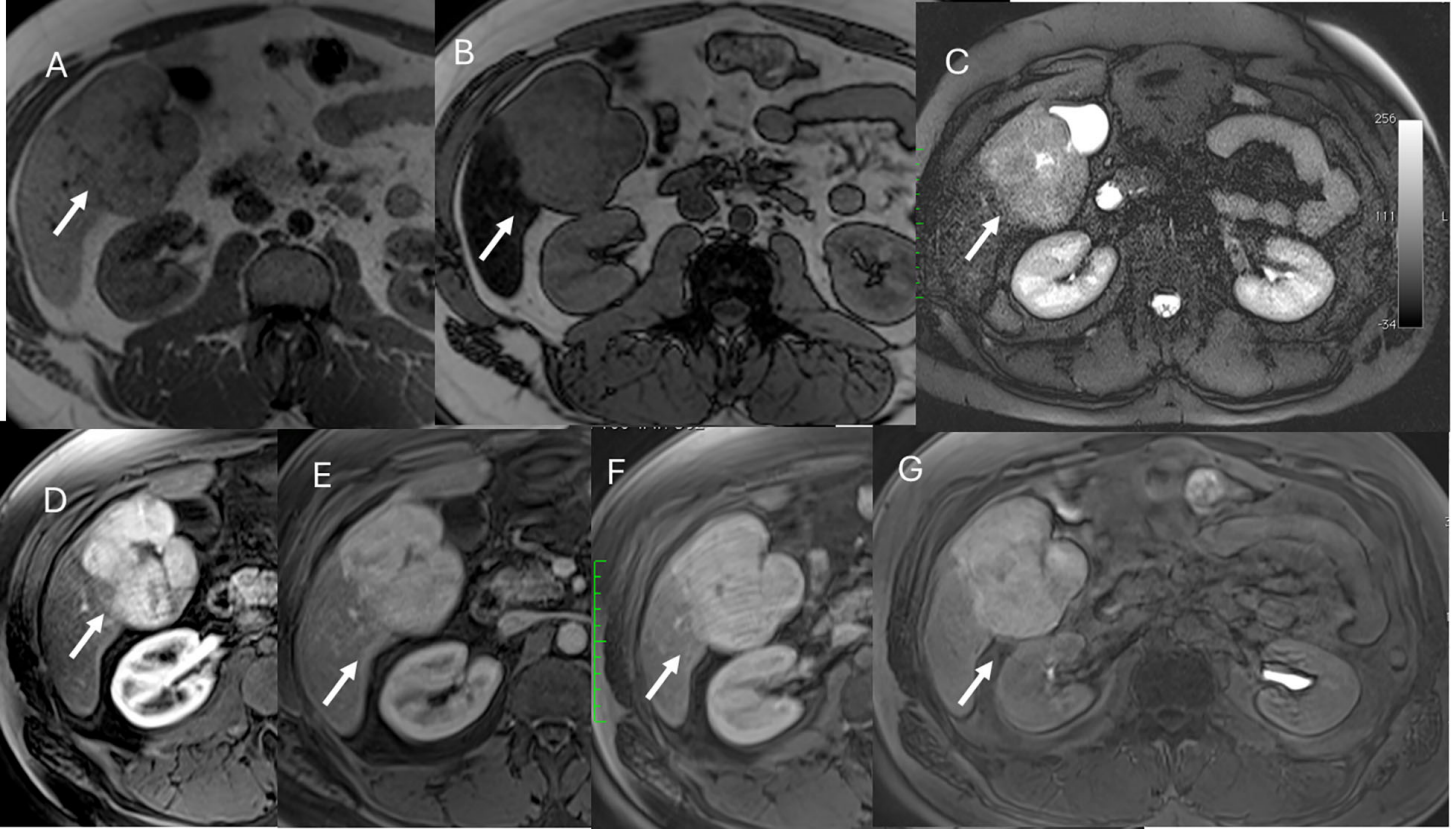
MRI assessment of FNH lesion (arrows) with Gd-EOB-DTPA contrast. In **(A)** (in-phase) and **(B)** (out-phase), the lesion shows hypointense signal with central scar and steatosis of the liver parenchyma in **(B)**. In C (T2-W FS sequence), the lesion is hyperintense with central scar. During contrast study, the lesion shows non-rim hyperenhancement during arterial phase **(D)**, no washout during portal **(E)** or transitional **(F)** phases, and hyperintense signal on hepatospecific phase **(G)**.

**Figure 8:**
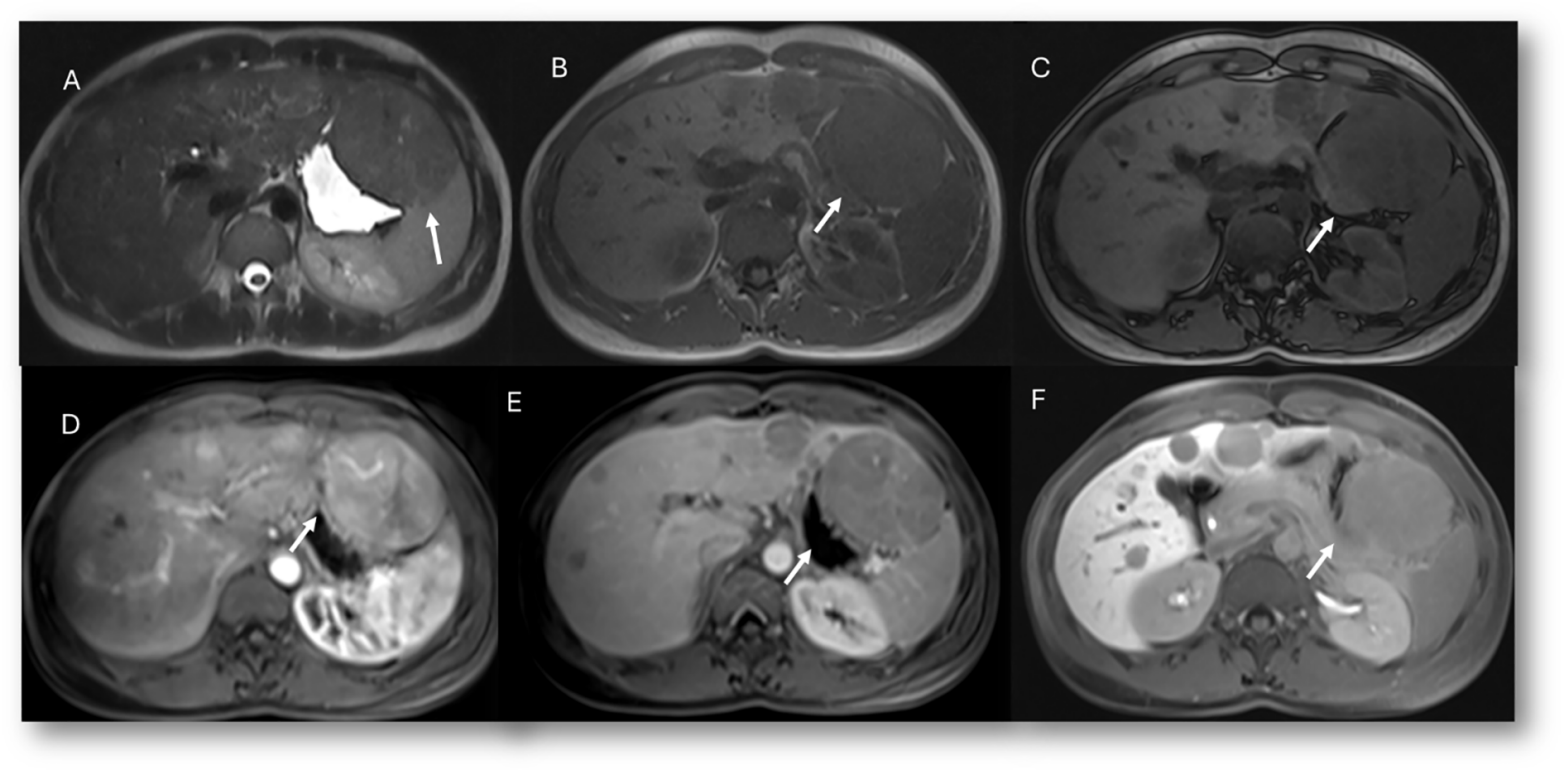
MRI assessment of multiple adenoma lesions (arrows) with Gd-EOB-DTPA contrast. In **(A)** (T2-W sequence), lesions are hyperintense; in **(B)** (T1-W in-phase) and **(C)** (T1-W out-of-phase), lesions are hypointense. During contrast study, lesions show non-rim hyperenhancement during arterial phase **(D)**, washout during portal phase **(E)**, and hypointense signal on hepatospecific phase **(F)**.

The established clinical indications for contrast-specific agents include characterization of focal lesions, detection and treatment planning for liver metastases, assessment of living liver donors (vascular and biliary anatomy and graft liver volume) or liver transplant complications, and study of bile leaks (94). With regard to characterization, it must be clear that hypointensity in the hepatobiliary phase is due to the lack of expression of OATP1B3, the principal contrast transporter. This pattern can be seen in both benign and malignant lesions such as hemangiomas and metastases, which is why hepatospecific contrast is not indicated when the diagnostic question is the differential diagnosis between these two lesions. In addition, diffuse uptake of contrast agent may be seen in focal nodular hyperplasia (FNH) and in FNH-like lesions, in liver parenchyma spared in steatosis patients, in some subtypes of adenomas (those with β-catenin activation), and in well-differentiated HCC (94). Since most adenomas do not show contrast retention, hepatospecific agents are suggested in the differential diagnosis between adenoma (**Figure 7**) and FNH lesions (**Figure 8**). Lastly, but of equal importance, is the fact that some lesions may present enhancement limited to the central portion due to retention of contrast by fibrous stroma, as in cholangiocarcinoma or in several types of liver metastases (95). In these conditions, it appears evident that lesion evaluation must necessarily be comprehensive and multiparametric, and not based only on the contrast pattern in the hepatospecific phase. However, bilirubin level evaluation remains mandatory, as hyperbilirubinemia can alter the results, and the MRI examination may not be diagnostic, since OATP1B3 is the principal contrast transporter, with optimal values being <6–8 mg/dl (96).

The evaluation of HCC deserves separate consideration. According to EASL guidelines (39), extracellular contrast agents should be favored over gadoxetic acid. The reasons are multiple, due to discrepancies in the literature between the accuracy of the two contrasts in HCC characterization. The vascular pattern during the dynamic study remains the determining factor (APHE, washout, and capsule appearance) for the characterization of HCC, while hypointensity in the hepatospecific phase is an additional but not principal finding. Since injection of gadoxetic acid is associated with an increased risk of transient respiratory motion artifacts in the arterial phase (occurring in 2.4%–18% of cases), this can reduce image quality. Thus, the risk of having a non-diagnostic arterial phase, and therefore a compromised diagnosis, is high. EASL guidelines report that ECA-MRI identified APHE in a significantly higher proportion of patients than CT (97.6% vs. 81.5%; p < 0.001) or HBA-MRI (89.5%; p = 0.002) (39).

So, when choosing a contrast agent for a liver study, it is necessary to know the clinical question, the patient’s clinical history, renal and hepatic function, and breath-hold capacity. For the characterization of a focal lesion, it is advisable to perform at least a T2W sequence, which can already assist in evaluating lesions such as hemangiomas or colorectal metastases, and then decide on the contrast.

### MRI study assessment and reporting

The liver MRI assessment must always be multiparametric (48, 97, 98). With regard to detection and characterization, it is necessary first to consider the conventional T2W and T1W sequences, supported by qualitative DWI analysis. Since even benign lesions may present diffusion restriction, ADC maps provide an aid in distinguishing benign from malignant lesions (99, 100). The evaluation of contrast phases remains crucial for lesion characterization (50, 101–104). Once a lesion has been identified, its location (segment and relationship with the capsule), size, vascular relationships (portal vein, inferior vena cava, and hepatic veins), relationships with the biliary branches, and extension to the hepatic hilum or extrahepatic structures must be defined. In patients with malignant lesions extending to the hilar region, particular attention must be paid to the relationship with the common hepatic duct; in this subgroup, it is appropriate to integrate the examination with cholangiographic sequences (105). Characterization of some lesions may be straightforward, as with cysts or HCC, while other non-typical lesions may require biopsy. However, it is always appropriate to direct the diagnosis toward benignity or malignancy to ensure proper patient management and follow-up. This process obviously requires careful clinical evaluation of the patient.

In staging, in addition to assessing the extent of disease within the liver, it is advisable to evaluate the lymph nodes of both the hepatic hilum and extrahepatic sites, as well as the peritoneum—for example, to exclude carcinomatosis in cholangiocarcinoma (106–108).

In the pre-surgical setting, it is necessary to evaluate the arterial and venous vascular anatomy of the liver parenchyma, as well as the anatomy of the biliary structures, including accessory vessels draining into the gallbladder.

In treatment evaluation, the analysis is strictly linked to the treatment itself. For conventional chemotherapy, dimensional variation is assessed. For targeted therapy, ablation, and radiotherapy, evaluation focuses on induced necrosis and the possible presence of residual disease. It is also necessary to assess treatment-related changes in hepatic parenchyma, as these impact liver function, and to report complications (109).

**Figure 9:**
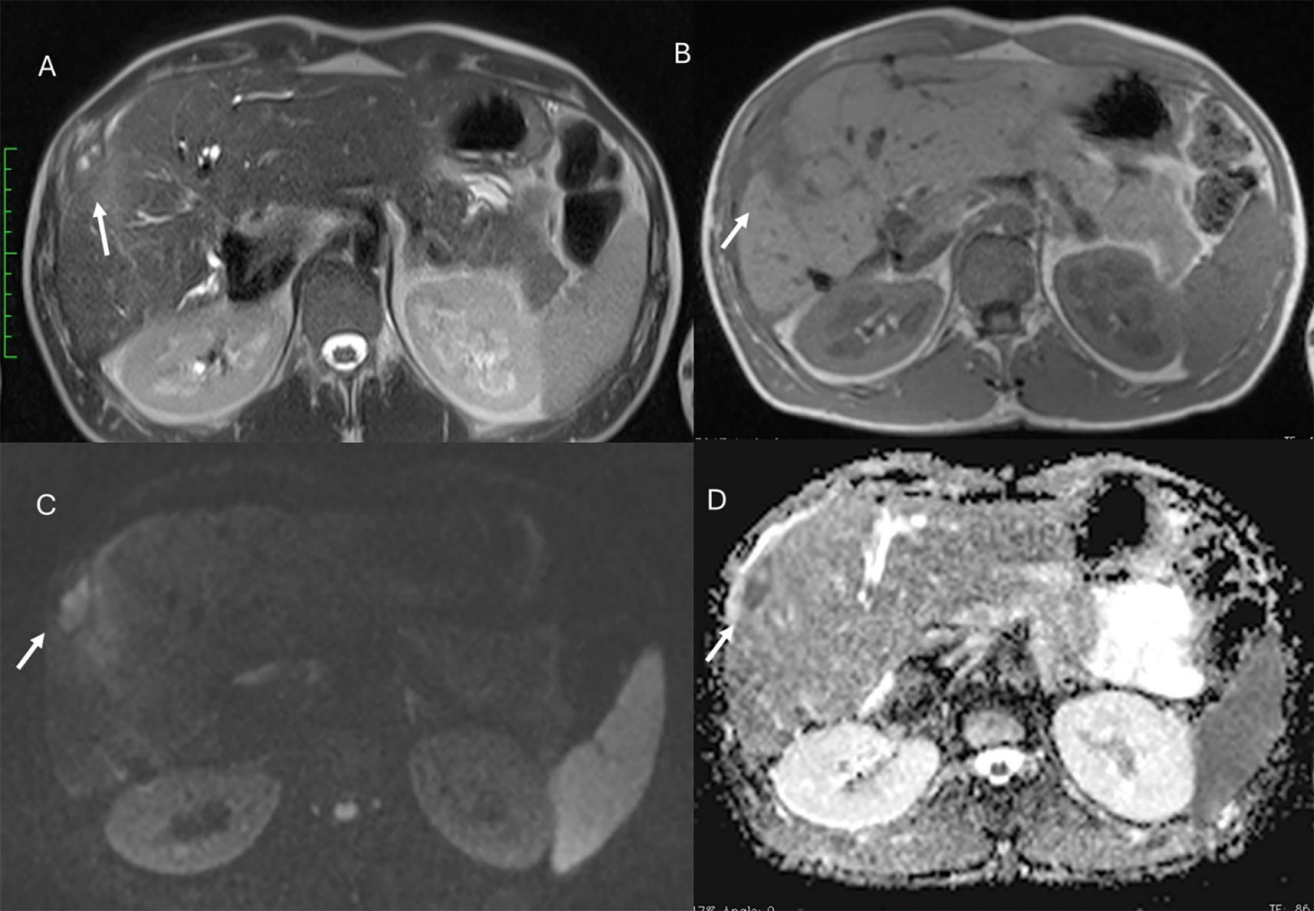
MRI of liver abscess after surgical resection. The lesion (arrows) shows hyperintense signal in T2-W sequence **(A)**, hypointense signal in T1-W sequence **(B)**, restricted diffusion on b = 800 s/mm² **(C)**, and hypointense signal on ADC map **(D)**. This demonstrates that a benign lesion can also show restricted signal and hypointensity on ADC maps.

**Figure 10:**
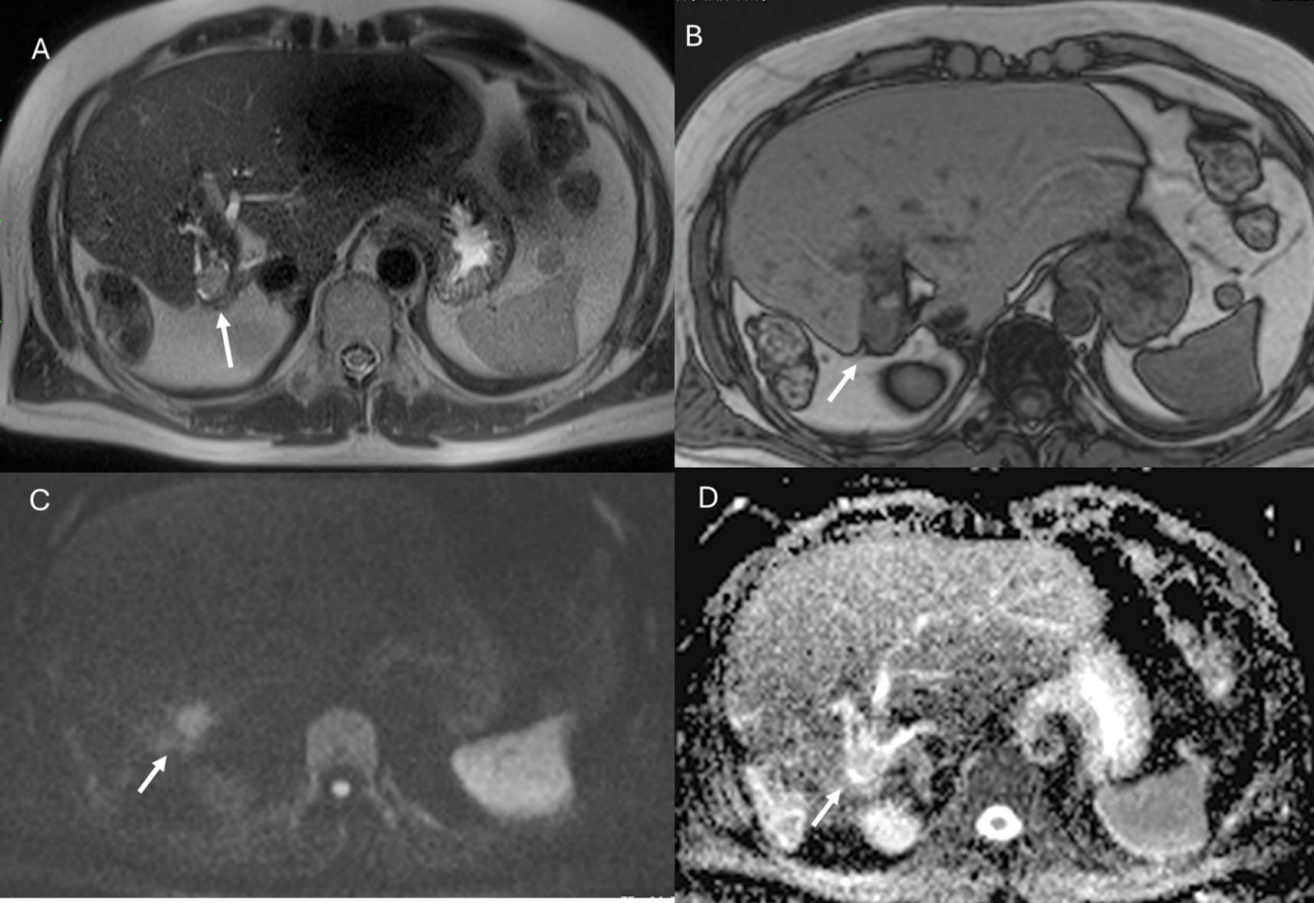
MRI assessment of residual disease after surgical resection. The lesion shows similar features to the patient in **Figure 9**, with hyperintense signal in T2-W sequence **(A)**, hypointense signal in T1-W out-of-phase sequence **(B)**, restricted diffusion on b = 800 s/mm² **(C)**, and hypointense signal on ADC map **(D)**. In this case, DWI is suggestive of residual lesion.

In the post-surgical setting, including transplantation, complications (**Figure 9**), technical success, new liver anatomy, presence of residual disease, or appearance of new lesions (**Figure 10**) must be evaluated. Attention must also be given to any extrahepatic findings (110).

Although liver MRI is a well-established modality with multiparametric capabilities, to fully exploit its potential it is mandatory to master the technique and optimize imaging protocols, apply advanced imaging concepts, and understand the use of different contrast media. Physiologic artifacts, although inherent to upper abdominal studies, can be minimized using triggering techniques and new motion-control strategies.

### Radiomics role in clinical practice

In recent years, the field of radiomics has witnessed remarkable evolution, driven by advances in computational power, artificial intelligence, and the development of sophisticated pattern recognition algorithms. These innovations have enabled the rapid, high-throughput extraction of quantitative data from standard medical imaging modalities such as CT, MRI, and PET. Unlike conventional radiological assessments that rely primarily on qualitative visual interpretation, radiomics deciphers subtle image patterns—often imperceptible to the human eye—that reflect underlying tumor biology. Through mathematical modeling and texture analysis, radiomics provides a non-invasive means to infer critical biological and molecular characteristics of tumors. This approach holds significant potential to enhance various aspects of cancer management, including improved diagnostic accuracy, more precise tumor grading and staging, early assessment of treatment response (particularly to chemotherapy or targeted therapies), and robust prediction of patient prognosis. By integrating radiomic features with clinical and pathological data, this methodology can offer personalized decision support, optimize treatment planning, and ultimately contribute to precision oncology (111–117).

Radiomics has emerged as a promising tool in liver oncology, particularly in the evaluation of colorectal liver metastases (CLM). By applying advanced machine learning and artificial intelligence techniques to conventional imaging modalities such as CT and MRI, radiomics enables the extraction of high-dimensional quantitative features that provide insights into tumor biology traditionally accessible only through invasive tissue sampling. Several studies have demonstrated the potential of radiomics in predicting key molecular and histopathological features, including RAS (KRAS/NRAS) and BRAF mutational status, microsatellite instability (MSI), and histological subtypes such as mucinous adenocarcinomas. These biomarkers have important implications for prognosis and treatment selection, particularly in guiding the use of anti-EGFR therapies or immunotherapy. Additionally, radiomic analysis has proven effective in characterizing histopathological growth patterns of liver metastases—namely desmoplastic, pushing, and replacement patterns—which are associated with distinct biological behaviors, therapeutic responses, and clinical outcomes. For example, desmoplastic growth patterns are linked to better overall survival and a more immunogenic microenvironment, while replacement patterns are correlated with resistance to systemic therapies. Despite its promise, routine clinical application of radiomics is still limited by technical challenges, such as variability in image acquisition, lack of standardized feature extraction protocols, and the need for external validation in multicenter studies. Nonetheless, ongoing research continues to refine these methods, bringing radiomics closer to integration into precision oncology workflows for liver metastases (2, 15–19, 103–105, 114, 115).

The integration of radiomics into clinical workflows has the potential to significantly improve personalized treatment strategies. For instance, identifying RAS wild-type status through imaging could help select patients for anti-EGFR therapies, while MSI detection could guide the use of immunotherapy. Additionally, radiomics-based assessment of histological subtype or growth pattern can stratify patients according to prognosis and expected response to chemotherapy, thus helping to avoid overtreatment in non-responders and tailor more aggressive interventions for high-risk patients (2, 15–19, 103–105, 114).

Several studies have validated the association between radiomic features and biological markers using large datasets and machine learning classifiers, achieving high predictive accuracy. Notably, combined models integrating radiomics with clinical data have outperformed models based on imaging or clinical features alone, reinforcing the value of a multimodal approach. However, despite these promising results, translation of radiomics into everyday clinical practice remains limited by several challenges (118–131).

Several challenges currently hinder the clinical translation of radiomics, with the most significant being the lack of standardization in image acquisition, processing protocols, and reconstruction algorithms. Inconsistent methodologies across imaging centers contribute to limited reproducibility and generalizability of results. Furthermore, many studies suffer from small sample sizes, lack of external validation cohorts, and inadequate management of class imbalance, all of which compromise the robustness of radiomics models (132–137).

A critical concern is the high dimensionality of radiomic features, which often leads to model overfitting, where a predictive model performs well on training data but fails to generalize to new, unseen cases. Overfitting typically results from including too many irrelevant or redundant features. Mitigation strategies include feature selection, dimensionality reduction, model regularization, and, importantly, validation using independent datasets. Conversely, underfitting can occur when the model is overly simplistic or unable to capture the complexity of the data, leading to poor performance even during training (132–137).

To ensure reliable and clinically meaningful outcomes, radiomics studies must incorporate high-quality imaging data, harmonized protocols, comprehensive and balanced datasets, and clearly defined validation frameworks. Performance metrics should extend beyond overall accuracy to include class-wise sensitivity and specificity, particularly in datasets with imbalanced outcomes. Ultimately, radiomics analysis must address these methodological issues to generate robust, reproducible, and generalizable models suitable for application across diverse patient populations (138–152).

Moreover, the limited interpretability of radiomic models remains a critical obstacle to their integration into clinical practice. Improving model transparency is imperative to foster clinician confidence and facilitate evidence-based decision-making. Only through such advancements can radiomics evolve from a promising research tool into a reliable component of personalized medicine (12, 111, 138–160).

## The power of artificial intelligence in imaging acquisition

The integration of AI into imaging acquisition represents one of the most transformative advancements in modern radiology. While the past decades have focused largely on improvements in scanner hardware, coil design, and pulse sequence optimization, the current era is characterized by the seamless embedding of AI-driven algorithms into nearly every stage of the image acquisition process. This shift is redefining both the efficiency and quality of diagnostic imaging, particularly in complex and high-resolution modalities such as liver MRI (8, 91, 92, 161–164).

Traditionally, MRI protocols have been highly operator-dependent, with scan prescription, sequence selection, and parameter adjustment relying heavily on technologist expertise. AI-based planning tools now enable automated detection of anatomical landmarks and pathology-relevant regions, allowing tailored sequence planning with consistent field-of-view alignment and optimal slice angulation (8). This standardization improves reproducibility between studies, which is critical in longitudinal liver lesion assessment and in ensuring the robustness of radiomics analyses (111–117).

Patient motion, whether from breathing, cardiac pulsation, or involuntary movement, remains one of the most significant challenges in abdominal imaging. AI-powered motion correction algorithms can identify and selectively reprocess corrupted k-space data, reducing the need for repeated acquisitions. DL-based reconstructions can denoise images while preserving critical fine detail, improving lesion conspicuity in low–signal-to-noise environments or under abbreviated protocols (8, 91, 92). Such algorithms have shown particular promise in enhancing arterial phase image quality in gadoxetate disodium–enhanced studies, where transient respiratory motion often limits diagnostic confidence (93, 94, 161–169).

Optimal timing of dynamic sequences is crucial for lesion characterization. AI-based bolus-tracking systems can detect subtle signal changes in real time, initiating acquisition at the precise moment of peak arterial enhancement (8). Unlike fixed-delay protocols, these adaptive methods compensate for patient-specific variations in cardiac output and circulation time, thereby improving consistency in vascular and perfusion imaging.

One of the most powerful contributions of AI is in image reconstruction. DL-accelerated techniques allow higher acceleration factors than conventional parallel imaging or compressed sensing alone, reducing acquisition times without sacrificing spatial resolution (8). This enables high-quality, multiphase imaging under a single breath-hold, an advancement particularly beneficial for patients with limited compliance, and mitigates the traditional trade-off between scan speed and image quality.

As abbreviated MRI protocols gain traction in liver cancer surveillance (32–35, 76), AI plays a pivotal role in ensuring diagnostic adequacy despite reduced sequence sets. Automated lesion detection, quality control, and phase selection can identify insufficient acquisitions in real time, prompting immediate reacquisition rather than delayed recall. This is particularly valuable in high-volume screening programs, where throughput must be balanced with diagnostic rigor (8, 91).

AI-enhanced acquisitions are not only visually superior but also quantitatively robust. By standardizing acquisition parameters and minimizing artifacts, AI ensures that extracted radiomic features are less influenced by technical variability. This stability improves the reproducibility and generalizability of predictive models, thereby accelerating the clinical translation of imaging biomarkers for prognosis, treatment response assessment, and personalized therapy planning (111–131).

Despite its promise, AI in imaging acquisition faces challenges, including the need for multi-vendor compatibility, rigorous external validation, and transparent algorithm design to maintain clinician trust (132–137). Furthermore, regulatory frameworks must evolve to accommodate continuously learning systems that adapt to evolving imaging protocols. Future developments will likely include AI agents capable of fully autonomous protocol selection, on-the-fly adjustment during scanning, and direct integration of acquisition data into decision-support platforms (8, 91).

In summary, AI-driven imaging acquisition is rapidly moving from experimental implementation to routine clinical practice. Its ability to optimize scan planning, improve image quality, and standardize data acquisition represents a critical step toward precision imaging, particularly in liver MRI, where subtle contrast patterns and small lesion detection are central to patient management.

## Conclusion

Magnetic resonance imaging (MRI) is currently recognized as the most suitable diagnostic tool for the detection and characterization of focal liver lesions. The combination of morphological and functional data allows, in different clinical scenarios, high diagnostic performance in characterizing even very small lesions and, therefore, improves patient management by reducing costs and time associated with inconclusive diagnostic tests. MRI should not be prescribed to all patients with focal liver lesions; the indications must be well defined (knowing when it is useful and when it may represent a waste of resources), and the characteristics of individual patients must be considered (since not all are fit for MRI examination). Equally important is the use of the most appropriate contrast medium in relation to the clinical question, which remains the responsibility of the radiologist.

An abbreviated protocol should be performed only if it provides the information necessary for correct patient management. Radiomics has emerged as a promising tool in liver oncology, particularly in the evaluation of colorectal liver metastases. However, the limited interpretability of radiomic models remains a critical obstacle to their integration into clinical practice. Improving model transparency is imperative to foster clinician confidence and support evidence-based decision-making. To fully realize the clinical value of radiomics, it is essential to address several methodological hurdles, including the standardization of image acquisition and analysis workflows and rigorous validation across large and diverse patient cohorts.
